# Does surgery modify growth kinetics of breast cancer micrometastases?

**DOI:** 10.1054/bjoc.2001.1969

**Published:** 2001-08

**Authors:** R Demicheli, P Valagussa, G Bonadonna

**Affiliations:** Istituto Nazionale Tumori via Venezian 1, Milano, 20133, Italy

**Keywords:** breast cancer, growth kinetics, micrometastases, surgery signals, tumour dormancy

## Abstract

Surgery should be considered as a major perturbing factor for metastasis development in laboratory animals. The different time distribution of mortality for 1173 patients undergoing mastectomy in comparison with 250 untreated patients suggests that primary tumour removal could result in changes of the metastatic process even for breast cancer. © 2001 Cancer Research Campaign http://www.bjcancer.com


*British Journal of Cancer* (2001) **85**(4), 490–492
© 2001 Cancer Research Campaign
doi: 10.1054/ bjoc.2001.1969, available online at http://www.idealibrary.com on IDEAL

http://www.bjcancer.com

Hypothesis

### Does surgery modify growth kinetics of breast cancer micrometastases?

**R Demicheli, P Valagussa and G Bonadonna**

Istituto Nazionale Tumori, via Venezian 1, 20133, Milano, Italy

**Summary** Surgery should be considered as a major perturbing factor for metastasis development in laboratory animals. The different time distribution of mortality for 1173 patients undergoing mastectomy in comparison with 250 untreated patients suggests that primary tumour removal could result in changes of the metastatic process even for breast cancer. © 2001 Cancer Research Campaign http://www.bjcancer.com

**Keywords**: breast cancer; growth kinetics; micrometastases; surgery signals; tumour dormancy

It was reported that in laboratory animals total (Gunduz et al, 1979) or partial (Braunschweiger et al, 1982) tumour removal may stimulate cell proliferation in macro-metastatic foci, due to an in serum detectable growth-stimulating factor (Fisher et al, 1989). More recently, the direct study of micro-metastases, proved that for some experimental tumours producing angiogenesis inhibitor factors, primary tumour removal caused a switch of micrometastatic foci to the angiogenic phenotype, resulting in growth of metastases (Holmgren et al, 1995). All these findings suggest that, at least in animal models, surgery should be considered as a major perturbing factor for metastasis development. No similar findings about surgery-induced tumour growth changes, if any, have been achieved in humans. Therefore, in spite of the very wide use of surgery to treat neoplasms, its effect on residual tumour growth dynamics is virtually unknown.

We report here an investigation on breast cancer mortality versus time in patients undergoing mastectomy alone as primary treatment and in patients not receiving any form of therapy. The comparison between the two mortality patterns suggests a possible influence of surgery on the course of the disease.

### MATERIALS AND METHODS

A total of 1173 patients who from 1964 through to 1980 entered into three different clinical trials at the Milan Cancer Institute, with mastectomy alone as primary treatment for operable breast cancer, were retrospectively evaluated. Primary tumour was treated by radical or modified radical mastectomy and no patient received postoperative radiotherapy or chemotherapy. After surgery, patients were examined every 6–8 months during the first 3 years and once a year thereafter. Treatment failure was defined as the first clinically documented evidence of new disease manifestation(s). A detailed description of patients and treatments is reported in Demicheli et al (1996).

*Received 26 March 2001*
*Revised 14 May 2001*
*Accepted 21 May 2001*

*Correspondence to:* R Demicheli

As a historical database of untreated patients, the series reported by Bloom et al (1962) was considered. A total of 250 patients with breast cancer, admitted to the Middlesex Hospital for terminal care, were studied. No patient was treated with any form of surgery, radiation therapy or hormone therapy. For a detailed description of patients the interested reader can consult the original paper.

From the published mortality data of the Bloom series and from our own data we calculated the yearly hazard rate of death. Our application involved a discretization of the time axis, and the timing of the death risk was studied by estimating the event-specific hazard rate as the conditional probability of dying in a time interval, given that the patient was alive at the beginning of the interval. The yearly discrete hazard of death was calculated by means of the life-table method (Statistical Sciences, 1995).

### RESULTS

The death-specific hazard rate for all patients is displayed in Figure 1. The curve obtained from the Bloom series shows a single surge, peaking at about the 4th–5th year, followed by a near constant plateau. On the contrary, the curve resulting from Milan data shows a double-peaked pattern, with a first major mortality surge reaching the maximum at the 3rd–4th year and a second well evident mortality increase near the 8th year. Moreover, the hazard rates of the Bloom series are quite higher than the corresponding values of the Milan series.

### DISCUSSION

For the Milan series, the time distribution of first treatment failure had been already reported to be double-peaked (Demicheli et al, 1996). The cause-specific hazard function for local-regional recurrences and distant metastases presented an early peak approximately at 18 months after surgery, a second peak at approximately 60 months with a plateau-like tail extending out to 15 years. This finding was confirmed by a similar analysis on a second series of 877 node positive patients receiving adjuvant CMF (Demicheli et al, 1999). Even a preliminary analysis of Milan mortality showed two peaks, with impressive quantitative and qualitative

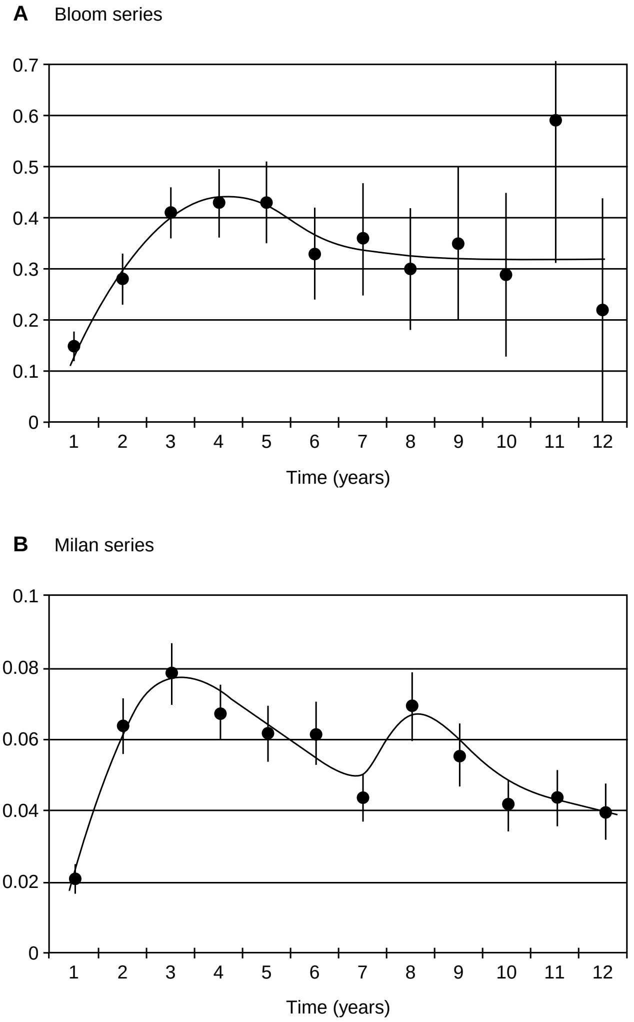

**Figure 1** Mortality of breast cancer patients: comparison between untreated patients (Bloom series) and patients undergoing mastectomy (Milan series). (**A**) Hazard function for death for 250 never treated women who eventually died. (**B**) Hazard function for 1173 women undergoing mastectomy alone as primary treatment, 625 of whom died similarity to another recently published series of surgically treated patients (Karrison et al, 1999; Demicheli et al, 2000). Therefore, the double-peaked pattern of breast cancer natural history (recurrence and mortality) after primary tumour removal should be considered as confirmed. A reasonable hypothesis to explain the observed patterns of the hazard functions is to assume that, as in animal models, primary tumour removal may result in sudden acceleration of metastatic process by stimulating tumour cell proliferation and/or angiogenesis (Demicheli et al, 1997). According to this view, multiple peaks should only appear for patients undergoing surgical treatment.

It is obvious that to compare surgery treated patients with untreated patients only retrospective studies could be performed. Therefore, the first step was to document the natural history of the untreated disease, in order to establish a baseline by which to judge the effects of surgery. Breast cancer has been considered a treatable disease for at least the last several hundred years. So, series of untreated but well-documented patients are uncommon. Among them most authors consider the Bloom series a reliable database with a fair degree of accuracy.

The most important result emerging from this study is the occurrence of two peaks in the hazard rate curve for the Milan series (patients who underwent mastectomy), and only one peak for the Middlesex series (untreated patients) (Figure 1).

Many caveats, a few of which are listed below, need to be considered when comparing the two curves. The Bloom series resulted from a retrospective investigation covering several decades. Death for breast cancer (verified by necropsy) was required as selection criteria for the studied patients, who were generally admitted to the hospital with advanced disease (74.4% stage IV, 23.2% stage III) for terminal care. As the duration of the disease was based on statements made by the patient or her relatives about the onset of the initial symptom (a lump in the breast in 83% of cases), the survival time is affected by a certain degree of uncertainty. Moreover, as all patients eventually died (although that required 16 years, remarkably), the hazard rate was elevated. Due to fluctuations in hazards towards the end of the observed time, we limited the analysis to 12 years.

Milan patients were prospectively recruited. Selection criteria consisted of histological confirmation of resectable unilateral breast cancer with no evidence of distant metastases. Following the disease recurrence some form of treatment was given (hormone therapy, chemotherapy, radiation therapy). Survival was defined from the date of mastectomy, disregarding the symptomatic time prior to surgery, and mortality included deaths for any cause. As at the time of analysis approximately 50% of patients were alive, Milan data show a lower hazard rate compared to the Bloom series.

Despite these concerns, we believe that, as more direct comparisons between treated and untreated patients are not available, the comparison between the curves obtained from Bloom and Milan data should be considered. The comparison gives some support to the hypothesis that in breast cancer surgical removal of the primary tumour may induce changes in the growth kinetics of metastatic foci, like in animal models. In our opinion a reasonable hypothesis, explaining the different pattern of death hazard functions, may be a multi-fold consequence of mastectomy, i.e. the ‘cure’ of a significant fraction of patients, and the change of the ‘natural’ recurrence and death timing for some of the others. If that is so, it will be relevant both to the screening policy and to the treatment strategy for breast cancer.

### REFERENCES

Bloom HJG, Richardson WW and Harries EJ (1962) Natural history of untreated breast cancer (1805–1933). *BMJ* **2**: 213–221

Braunschweiger PG, Schiffer LM and Betancourt S (1982) Tumour cell proliferation and sequential chemotherapy after partial tumour resection in C3H/HeJ mammary tumours. *Breast Cancer Res Treat* **2**: 323–329

Demicheli R, Abbattista A, Miceli R, Valagussa P and Bonadonna G (1996) Time distribution of the recurrence risk for breast cancer patients undergoing mastectomy: further support about the concept of tumour dormancy. *Breast Cancer Res Treat* **41**: 177–185

Demicheli R, Miceli R, Brambilla C; Ferrari L, Moliterni A, Zambetti M, Valagussa P and Bonadonna G (1999) Comparative analysis of breast cancer recurrence risk for patients receiving adjuvant Cyclophosphamide, Methotrexate, Fluorouracil (CMF). Data supporting the occurrence of ‘cures’. *Breast Cancer Res Treat* **53**: 209–215

Demicheli R, Miceli R, Valagussa P and Bonadonna G (2000) Re: Dormancy of mammary carcinoma after mastectomy. *J Natl Cancer Inst* **92**: 347–378

Demicheli R, Retsky MW, Swartzendruber DE and Bonadonna G (1997) Proposal for a new model of breast cancer metastatic development. *Ann Oncol* **8**: 1075–1080

© 2001 Cancer Research Campaign 

Fisher B, Gunduz N, Coile J et al (1989) Presence of a growth-stimulating factor in serum following primary tumour removal in mice. *Cancer Res* **49**: 1996–2001

Gunduz N, Fisher B and Saffer EA (1979) Effect of surgical removal on the growth and kinetics of residual tumour. *Cancer Res* **39**: 3861–3865

Holmgren L, O'Reilly MS and Folkman J (1995) Dormancy of micrometastases: balanced proliferation and apoptosis in the presence of angiogenesis suppression. *Nature Med* **1**: 149–153

Karrison TG, Ferguson DJ and Meier P (1999) Dormancy of mammary carcinoma after mastectomy. *J Natl Cancer Inst* **91**: 80–85

Statistical Sciences, S-PLUS Guide to Statistical and Mathematical Analysis, Version 3.3, Seattle: StatSci a Division of MathSoft, Inc., 1995

 *© 2001 Cancer Research Campaign*

